# Rockwood Type VIb Acromioclavicular Joint Dislocation With Ipsilateral Proximal Humerus Fracture-Dislocation and Scapular Spine Fracture: A Case Report

**DOI:** 10.7759/cureus.107156

**Published:** 2026-04-16

**Authors:** Stanzin Sonam, Anant Krishna, Amul Aggarwal

**Affiliations:** 1 Orthopaedics, Atal Bihari Vajpayee Institute of Medical Sciences and Dr. Ram Manohar Lohia Hospital, New Delhi, IND

**Keywords:** acromioclavicular joint dislocation, coracoclavicular screw, proximal humerus fracture-dislocation, rockwood type vi, scapular spine fracture, shoulder girdle injury

## Abstract

Rockwood type VI acromioclavicular (AC) joint dislocations are extremely rare high-energy injuries characterized by inferior displacement of the distal clavicle and are often associated with complex shoulder girdle injury. We report the case of a 36-year-old male who sustained a Gustilo-Anderson type IIIA open Rockwood type VIb AC joint dislocation associated with a Neer two-part proximal humerus fracture-dislocation and an ipsilateral scapular spine fracture following a road traffic accident. The patient underwent early surgical debridement and single-stage operative management, including open reduction and internal fixation of the proximal humerus using a locking plate (Proximal Humerus Multiple Locked Plate; Sharma Orthopedic, Gujarat, India) and stabilization of the AC joint with a coracoclavicular screw (Cortical Screw 3.5 mm; Sharma Orthopedic, Gujarat, India), while the scapular fracture was managed conservatively. At the 12-month follow-up, the patient demonstrated excellent radiological and functional outcomes, without complications. This case highlights the importance of a high index of suspicion for associated injuries in high-grade AC joint dislocations and shows that early, single-stage surgical stabilization using sound biomechanical principles can restore anatomy and function even in rare and complex injury patterns.

## Introduction

Acromioclavicular (AC) joint injuries are a frequent consequence of high-energy trauma to the shoulder and represent approximately 10% of all injuries involving the shoulder girdle [1,2]. Among the injury patterns described in the Rockwood classification, which range from minor sprains (type I) to severe displacement injuries (type VI), type VI injuries are exceptionally uncommon [3]. These injuries are defined by inferior displacement of the distal clavicle either beneath the acromion (subacromial) or the coracoid process (subcoracoid), reflecting severe disruption of the stabilizing structures of the AC joint. Since their first description by Patterson [4] in 1967, only a limited number of such cases have been documented in the orthopedic literature to date, including reports of both subacromial (type VIa) and subcoracoid (type VIb) variants [1].

High-grade AC joint disruptions (Rockwood types IV-VI) are characterized by complete rupture of the AC and coracoclavicular (CC) ligaments, along with compromise of the delto-trapezial fascia, which are the major soft tissues stabilizing the clavicle to the scapula. Because of the substantial instability produced by these injuries and the potential for persistent functional impairment, operative stabilization is generally recommended [5].

Proximal humerus fractures represent the most common fractures of the shoulder girdle. However, their occurrence in combination with AC joint disruption is exceedingly rare. Chuaychoosakoon and Klabklay reported the first documented case of this injury combination in 2020, involving a Neer four-part proximal humerus fracture associated with a Rockwood type III AC joint disruption, which was managed surgically [6]. Scapular fractures are relatively uncommon, accounting for only 0.4-1% of all fractures, and are typically associated with high-energy trauma [7]. Together, these injuries may represent a complex shoulder girdle injury pattern resulting from high-energy force transmission.

We report the case of a 36-year-old male who sustained a Gustilo-Anderson type IIIA open Rockwood type VIb AC joint disruption associated with a Neer two-part proximal humerus fracture-dislocation and an ipsilateral scapular spine fracture following a high-energy road traffic accident. The AC joint disruption and proximal humerus fracture were successfully treated operatively, while the scapular spine fracture was managed non-operatively. To our knowledge, this combination of injuries has not been previously reported.

## Case presentation

History

A 36-year-old, right-hand-dominant male presented to the emergency department following a high-energy road traffic accident. The patient reported that he was struck on the right shoulder by the bucket of an excavator while he was driving a scooter, with a secondary impact resulting in extreme abduction and external rotation of the shoulder combined with a direct superior-to-inferior compression force. The patient was rushed to our emergency department within 30 minutes of his injury with severe right shoulder pain and swelling, with ecchymosis over the lateral shoulder and superior scapular region.

Clinical examination

The patient was hemodynamically stable. A clean lacerated wound measuring approximately 10 × 3 × 2 cm was noted over the anterior shoulder region extending from the junction of the middle and lateral third of the clavicle toward the axilla, with exposure of clavipectoral fascia, pectoralis muscle, delto-trapezial fascia, tip of coracoid with conjoint tendon, and lateral end of clavicle. The shoulder exhibited gross deformity with loss of normal contour, and the arm was held in adduction. The patient was able to move his fingers, wrist, and elbow, and was able to feel light touch. The radial pulse was palpable, and the hand was warm with good capillary refill. Shoulder movements could not be assessed due to pain. There was marked tenderness over the proximal humerus, AC joint, and scapular region.

Comprehensive trauma evaluation, including clinical assessment by general surgery and neurosurgery teams, revealed no additional injuries, and Extended Focused Assessment with Sonography for Trauma demonstrated no evidence of free intraperitoneal or pericardial fluid and no sonographic signs of pneumothorax or hemothorax. These findings were documented at the time of initial trauma evaluation; however, representative images were not retained. The wound was thoroughly irrigated and dressed under aseptic conditions, and intravenous first-generation cephalosporin was administered.

Imaging

**Figure 1:**
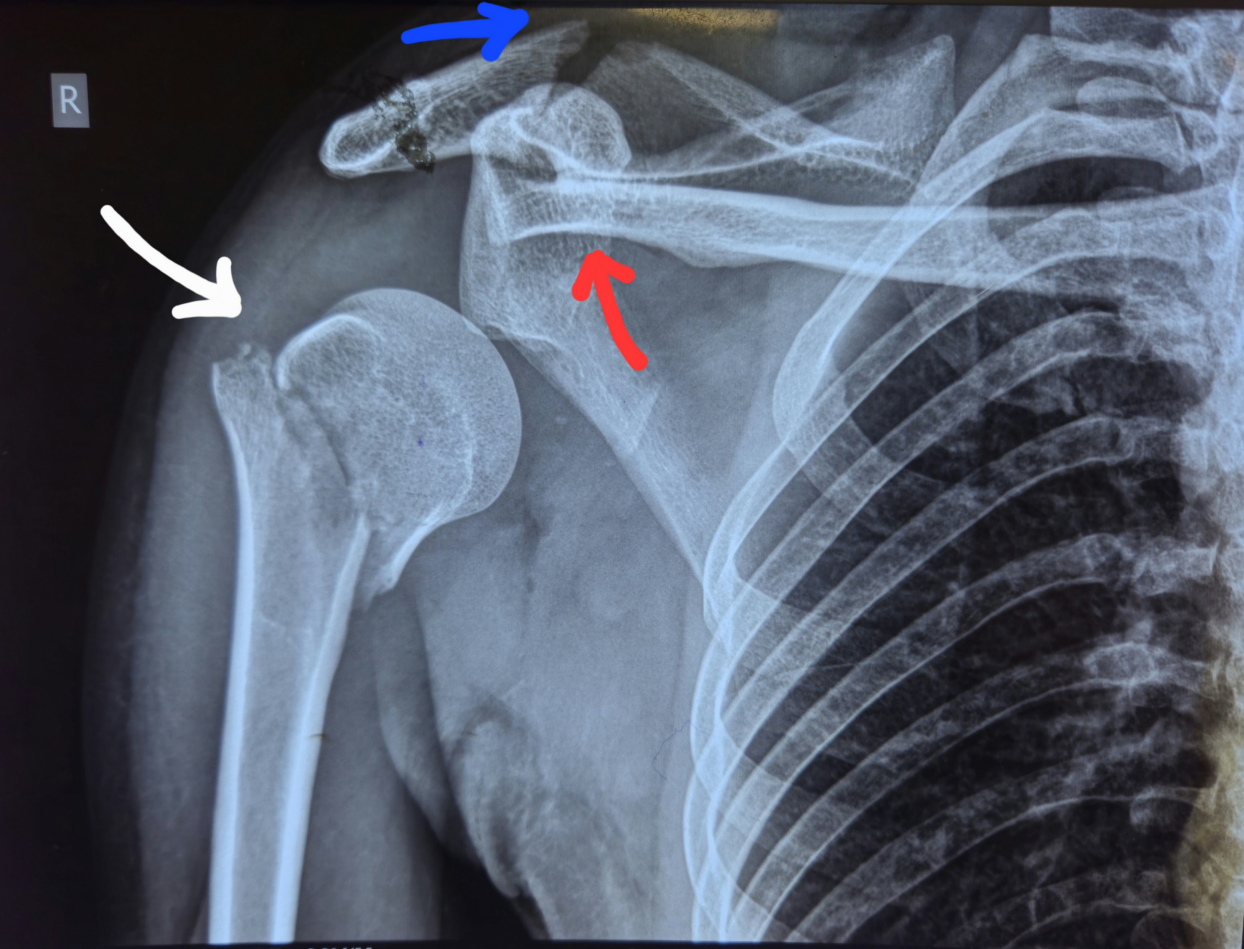
Preoperative radiograph of the affected shoulder demonstrating a displaced Neer two-part proximal humerus fracture with anterior-inferior humeral head dislocation (white arrow), ipsilateral Rockwood type VIb (subcoracoid) acromioclavicular joint dislocation (red arrow), and minimally displaced scapular spine fracture (blue arrow).

Anteroposterior radiographs of the affected shoulder demonstrated a displaced two-part proximal humerus fracture through the surgical neck with anterior-inferior dislocation of the humeral head, an ipsilateral AC joint dislocation with the distal clavicle positioned beneath the coracoid, and a minimally displaced scapular spine fracture (Figure 1).

Key diagnostic features included: (1) inferior displacement of the distal clavicle beneath the coracoid process; (2) associated proximal humerus fracture-dislocation involving the surgical neck; (3) open anterior shoulder wound with exposure of the coracoid process and surrounding soft-tissue structures; and (4) high-energy mechanism with forced abduction-external rotation and compressive forces.

Definitive management

Within four hours of injury, the patient underwent surgical debridement, open reduction of the dislocated joints, and definitive fracture fixation under general anesthesia. The patient was positioned in the beach-chair position with a bolster placed in the interscapular region. The right upper limb was draped separately to facilitate intraoperative limb manipulation.

A deltopectoral approach was used with the proximal part of the incision merging with the lacerated wound. Intraoperatively, the humeral head was found to be dislocated anteriorly, lying at the level of the anteroinferior glenoid rim, with an associated fracture of the proximal humerus through the surgical neck. The lateral end of the clavicle was identified beneath the coracoid process and posterior to the intact conjoint tendon, consistent with a type VIb AC dislocation, along with complete disruption of both the AC and CC ligaments and intact coracoacromial ligament. The delto-trapezial fascia was noted to be torn.

Open reduction of the humeral head dislocation was performed, followed by reduction of the proximal humerus fracture, which was provisionally stabilized using multiple Kirschner wires (Sharma Orthopedic, Gujarat, India). Intraoperative fluoroscopy confirmed anatomic reduction, with restoration of humeral head alignment relative to the glenoid and an appropriate neck-shaft angle. Fluoroscopic images were utilized intraoperatively but were not archived. Definitive fixation was then achieved using a proximal humerus internal locking system (Proximal Humerus Multiple Locked Plate; Sharma Orthopedic, Gujarat, India) applied to the anterolateral aspect of the proximal humerus, positioned approximately 5 mm distal to the greater tuberosity and 4 mm posterior to the bicipital groove. The surgical approach was then extended proximally by approximately 3 cm from the level of the coracoid process to address the AC joint disruption. Manual reduction restored the distal clavicle to its anatomical position superior to the coracoid process, and stabilization was achieved with the CC screw (Cortical Screw 3.5 mm; Sharma Orthopedic, Gujarat, India). The AC ligament and delto-trapezial fascia were carefully reconstructed using No.0-vicryl (polyglactin 910; Ethicon, Somerville, NJ, USA). The wound was then closed in layers. Postoperative intravenous first-generation cephalosporin was administered for three days.

The scapular spine fracture was managed conservatively with initial sling immobilization and analgesics, followed by early gentle range of motion exercises initiated at two weeks postoperatively. Progressive mobilization proceeded through structured physiotherapy, including pendulum exercises for the next two weeks, followed by passive and active range of motion exercises under the guidance of a physiotherapist. At eight weeks of follow-up, the patient demonstrated a near-normal range of motion, with mild pain at the terminal range of passive movement. By 12 weeks postoperatively, the patient was pain-free and had regained a near-normal range of motion. At this stage, a structured rehabilitation program focusing on rotator cuff and periscapular muscle strengthening was initiated, and the patient was allowed to return to work. At six months of follow-up, the patient had a pain-free range of motion with full strength of the shoulder girdle musculature.

Outcome

**Figure 2:**
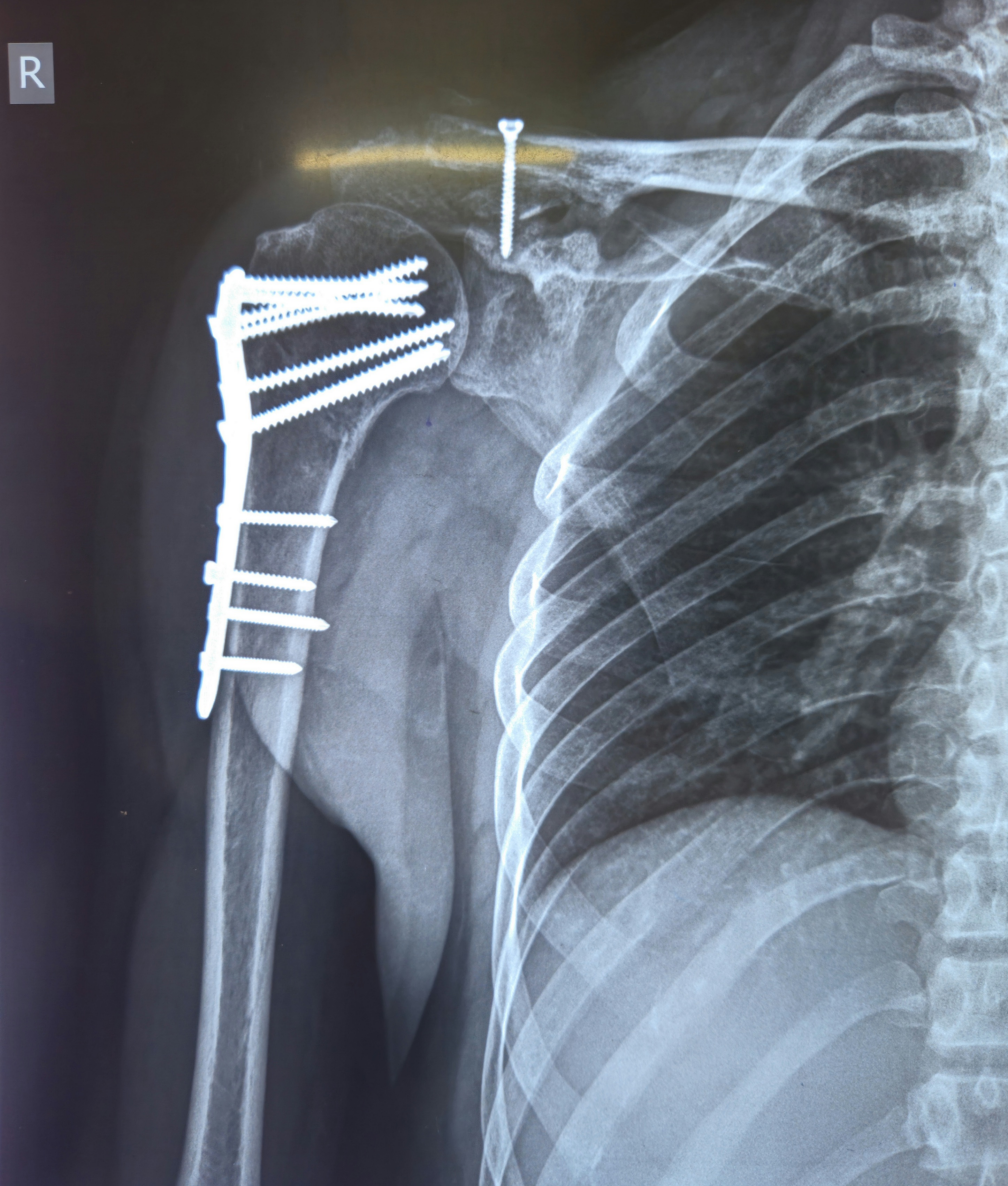
Postoperative radiograph of the affected shoulder at the 12-month follow-up showing complete union of the proximal humerus fracture with intact implant, maintained reduction of the glenohumeral joint and acromioclavicular joint stabilized with a coracoclavicular screw, and a healed scapular spine fracture.

At the 12-month follow-up, radiographs demonstrated union of the proximal humerus fracture with maintained anatomic alignment and intact hardware. The AC joint maintained a stable reduction with normal CC distance. The screw remained intact with no evidence of AC joint osteoarthritis. The scapular spine fracture had also achieved complete union with acceptable alignment (Figure 2).

**Figure 3:**
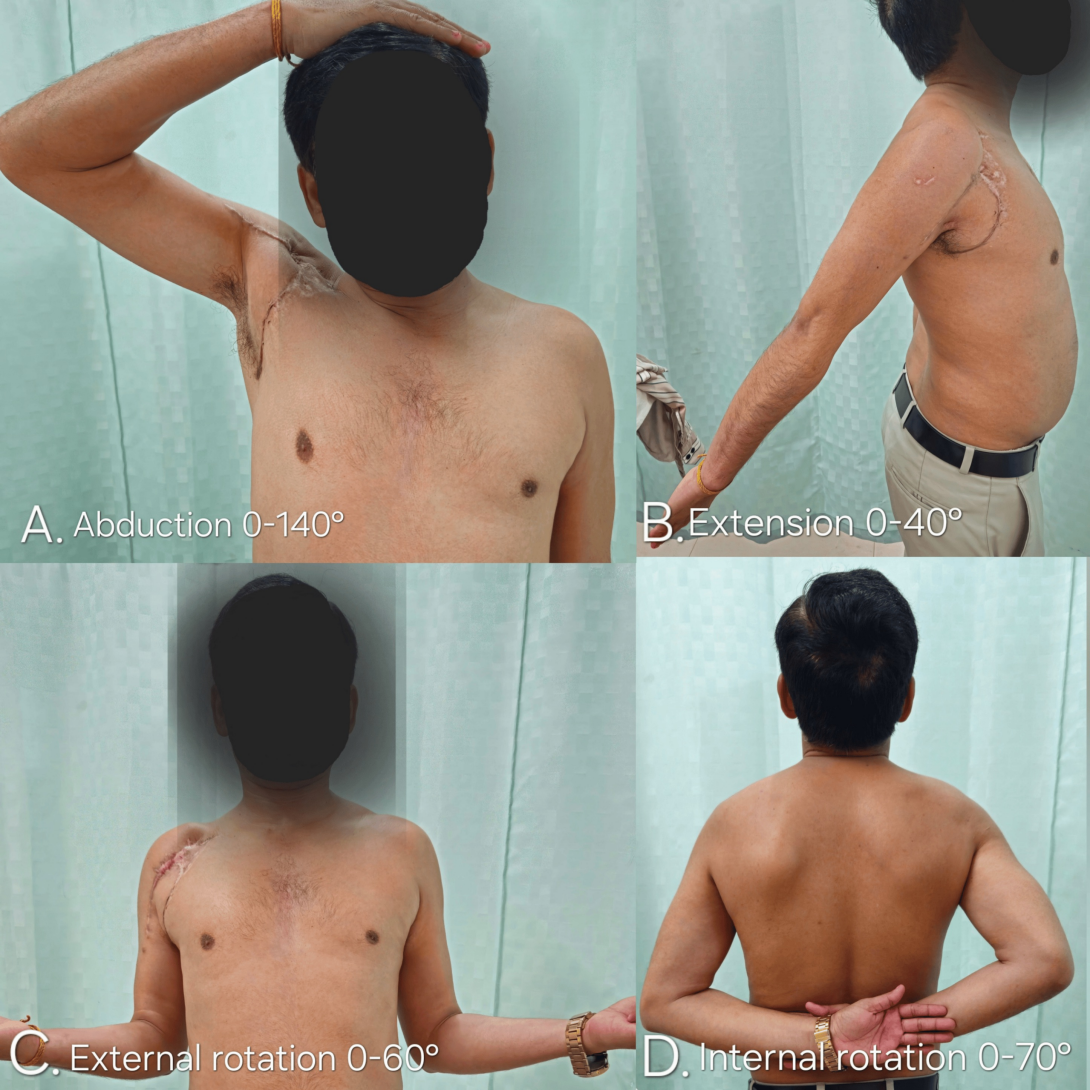
Clinical photographs at the 12-month follow-up demonstrating full functional recovery of the right shoulder with a near-normal range of motion, including (A) abduction, (B) extension, (C) external rotation, and (D) internal rotation.

Clinically, the patient achieved a functional, pain-free range of motion of the shoulder (Figure 3). Range of motion measurements were near normal when compared with the contralateral shoulder (Table 1). The surgical incision site and the laceration wound had healed completely, with satisfactory scars, no signs of infection, and no wound-related complications.

**Table 1:** Range of motion of both shoulder joints at the 12-month follow-up.

Range of motion	Right shoulder (injured side)	Left shoulder (normal side)	Percentage of normal
Forward flexion	0°–145°	0°–160°	90.6%
Extension	0°–40°	0°–40°	100%
Abduction	0°–140°	0°–155°	90.3%
Adduction	0°–40°	0°–50°	80%
External rotation	0°–60°	0°–70°	85.7%
Internal rotation	0°–70°	0°–70°	100%

Manual muscle testing demonstrated full strength of the shoulder girdle musculature, with all muscles graded 5/5 according to the Medical Research Council scale [8]. Functional evaluation revealed excellent outcomes, with a Constant-Murley score [9] of 92 and a Disabilities of the Arm, Shoulder and Hand score of 7 [10]. The patient expressed high satisfaction with no pain during daily activities and successfully returned to his pre-injury occupation without restrictions. No postoperative complications were observed. The chronological sequence of clinical events, management, and follow-up is summarized in Table 2.

**Table 2:** Timeline of clinical events from injury to final follow-up.

Time from injury	Event
0 hours	High-energy road traffic accident
0–0.5 hours	Arrival at the emergency department, initial assessment, imaging
0–4 hours	Wound lavage, antibiotics, surgical planning
4 hours	Definitive surgery: open reduction and internal fixation of the proximal humerus fracture dislocation + coracoclavicular screw fixation acromioclavicular joint dislocation
Postoperative day 1–3	Intravenous antibiotics, sling immobilization
2 weeks	Initiation of mobilization (structured physiotherapy)
12 weeks	Return to work
6–12 months	Full functional recovery and radiological union

## Discussion

Rockwood type VI AC joint dislocations are among the rarest AC joint injuries, with only 24 cases reported in the literature to date, including 10 type VIa and 14 type VIb injuries [1]. The rarity of this injury pattern limits the availability of high-level evidence, and current understanding is derived primarily from isolated case reports and small case series.

These injuries typically result from high-energy trauma, most commonly motor vehicle accidents [4,11-16]. This pattern is consistent with our case and reinforces the concept that type VI injuries reflect substantial force transmission to the shoulder girdle. The previously reported cases also demonstrate a high incidence of associated injuries, particularly ipsilateral clavicle fractures [13,17-19], scapular fractures [2,14-17,20], and brachial plexus injuries [12,17]. Our case similarly involved a complex injury combination of open Rockwood type VIb AC joint disruption with ipsilateral proximal humerus fracture-dislocation and scapular spine fracture, supporting the observation that type VI AC dislocations rarely occur in isolation and should prompt careful assessment for concomitant shoulder girdle and neurovascular injuries.

From a biomechanical perspective, the unique combination of injuries observed in this case can be explained by the direction and magnitude of force vectors acting on the shoulder girdle. Forced abduction and external rotation of the shoulder, combined with a direct superior-to-inferior compressive force applied to the acromion, likely resulted in inferior displacement of the distal clavicle beneath the coracoid process, producing a Rockwood type VIb AC dislocation. Simultaneously, this position would have generated a levering effect at the glenohumeral joint, leading to anterior-inferior dislocation of the humeral head with an associated surgical neck fracture. In addition, transmission of residual force across the scapular body and spine may have resulted in the associated scapular spine fracture. This combination of vertically directed compression and rotational forces highlights the complex biomechanical environment required to produce such a rare injury pattern.

Intraoperative findings across reported cases consistently show disruption of the AC and CC ligaments, while the coracoacromial ligament is usually preserved [4,11,17]. Given the severity of ligamentous disruption and the inherent instability of this injury, operative management is generally recommended. A variety of fixation methods have been described, including Kirschner wires (K-wires), CC screws, plate fixation, and distal clavicle resection. No single technique has emerged as the gold standard, and treatment is usually individualized according to associated injuries and surgeon preference [1].

The choice of fixation in this case was guided by multiple factors, including injury severity, associated injuries, resource availability, and surgeon familiarity. Given the extensive ligamentous disruption, open nature of the injury, and associated proximal humerus fracture-dislocation, rigid fixation with a CC screw was preferred to ensure reliable maintenance of reduction during the early healing phase. This approach is supported by prior reports by Patterson [4], Gerber and Rockwood et al. [11], and Emami et al. [20], in which CC screw fixation achieved satisfactory maintenance of reduction and favorable outcomes. In resource-limited healthcare settings and in the presence of patient-related financial constraints, CC screw fixation may remain a cost-effective and widely accessible alternative to modern suspensory fixation systems, which may be prohibitively expensive in many developing countries. Alternative techniques, such as suture-button fixation and arthroscopic stabilization, although less invasive, may not provide equivalent initial rigid fixation in the setting of severe soft-tissue compromise and complex fracture patterns, while hook plate fixation carries risks of subacromial impingement and requires secondary surgery for implant removal. Importantly, reported cases of CC screw fixations have demonstrated a low rate of recurrent instability when adequate stabilization is achieved, suggesting that long-term joint stability can be reliably maintained regardless of fixation method. However, the need for a secondary procedure for implant removal remains a recognized limitation [4,11,20].

The timing of surgery varied widely across the reported cases, ranging from acute intervention to delayed fixation performed several weeks or even months after injury. Although early stabilization is generally preferred to facilitate reduction and reduce soft-tissue contracture, satisfactory outcomes have also been achieved with delayed surgery, as seen in cases such as Patterson [4] and Neumann et al. [12]. Nevertheless, early intervention, as performed in our patient, likely improved the ease of reduction and contributed to the excellent functional recovery.

The concomitant occurrence of a proximal humerus fracture-dislocation with an AC joint injury is exceedingly uncommon. To date, only one such case has been reported in the literature, involving a lower-grade Rockwood type III AC joint injury associated with a proximal humerus fracture [6]. Accordingly, the present case represents the second documented instance of this combined injury pattern and, to our knowledge, the first reported case of a Rockwood type VI AC joint dislocation occurring in conjunction with a proximal humerus fracture-dislocation. The open nature of the injury further distinguishes this case and increases the risk of infection and soft-tissue complications, necessitating meticulous surgical management.

More than 90% of scapular fractures are managed conservatively, with excellent to good outcomes reported in up to 86% of cases, particularly when early mobilization is initiated [7]. However, in the setting of complex shoulder girdle injuries, the overall management strategy must be individualized. In such scenarios, surgical stabilization of the primary destabilizing injuries facilitates restoration of shoulder biomechanics and allows safe early mobilization, which is critical for optimizing functional recovery. In our case, although the scapular fracture itself did not require operative fixation, stabilization of the AC joint and proximal humerus enabled a structured rehabilitation protocol while minimizing the risk of secondary displacement and stiffness.

Outcomes in the reported cases demonstrate generally favourable results following surgical management, despite variability in fixation methods [4,11,19]. The favorable clinical and radiological outcome observed in our patient, characterized by proximal humerus fracture union, maintained AC joint reduction, and 80-100% recovery of shoulder range of motion compared to the contralateral side, indicating near-normal shoulder function, parallels these findings. Importantly, the absence of complications such as recurrent instability, avascular necrosis of the humeral head, AC joint arthritis, or hardware-related failure in our case further supports the principle that early anatomical reduction combined with stable, construct-appropriate fixation can successfully restore both vertical and horizontal stability, even in complex, multi-structural injuries.

Overall, this case expands the existing literature by illustrating a more severe and biomechanically complex variant of type VIb AC joint dislocation. It underscores the importance of systematic evaluation of the entire shoulder girdle in high-energy trauma and highlights that a tailored, single-stage surgical approach can achieve excellent functional and radiological outcomes. However, the inherent limitations of a single case report must be acknowledged, including potential observer and reporting bias, as outcomes were assessed clinically without blinding or comparative analysis. Therefore, while the outcome in this patient was excellent, it may not be generalizable to all similar injury patterns. Nevertheless, this case provides valuable insight into the management of rare and complex injury patterns.

## Conclusions

Rockwood type VI AC joint dislocations are rare, high-energy injuries frequently associated with complex shoulder girdle trauma. This case represents an exceptionally uncommon combination of a type VIb AC joint dislocation with a proximal humerus fracture-dislocation and scapular spine fracture. It emphasizes the importance of maintaining a high index of suspicion and performing a comprehensive clinical and radiological evaluation to identify associated injuries. In this case, early, single-stage surgical management with stable fixation successfully restored anatomical alignment, enabled early mobilization, and resulted in excellent functional and radiological outcomes. Such an approach may be effective in selected patients with similar complex injury patterns. CC screw fixation appears to be a practical and accessible option, particularly in resource-constrained settings. Further studies are required to validate these findings and establish optimal management strategies for such rare injury patterns.
